# An alternative mode of epithelial polarity in the *Drosophila* midgut

**DOI:** 10.1371/journal.pbio.3000041

**Published:** 2018-10-19

**Authors:** Jia Chen, Aram-Christopher Sayadian, Nick Lowe, Holly E. Lovegrove, Daniel St Johnston

**Affiliations:** The Gurdon Institute and the Department of Genetics, University of Cambridge, Cambridge, United Kingdom; The Francis Crick Institute, UNITED KINGDOM

## Abstract

Apical–basal polarity is essential for the formation and function of epithelial tissues, whereas loss of polarity is a hallmark of tumours. Studies in *Drosophila* have identified conserved polarity factors that define the apical (Crumbs, Stardust, Par-6, atypical protein kinase C [aPKC]), junctional (Bazooka [Baz]/Par-3), and basolateral (Scribbled [Scrib], Discs large [Dlg], Lethal [2] giant larvae [Lgl]) domains of epithelial cells. Because these conserved factors mark equivalent domains in diverse types of vertebrate and invertebrate epithelia, it is generally assumed that this system underlies polarity in all epithelia. Here, we show that this is not the case, as none of these canonical factors are required for the polarisation of the endodermal epithelium of the *Drosophila* adult midgut. Furthermore, like vertebrate epithelia but not other *Drosophila* epithelia, the midgut epithelium forms occluding junctions above adherens junctions (AJs) and requires the integrin adhesion complex for polarity. Thus, *Drosophila* contains two types of epithelia that polarise by fundamentally different mechanisms. This diversity of epithelial types may reflect their different developmental origins, junctional arrangement, or whether they polarise in an apical–basal direction or vice versa. Since knock-outs of canonical polarity factors in vertebrates often have little or no effect on epithelial polarity and the *Drosophila* midgut shares several common features with vertebrate epithelia, this diversity of polarity mechanisms is likely to be conserved in other animals.

## Abstract 2

A developmental study in the fruit fly Drosophila melanogaster shows that apical-basal polarity in the midgut epithelium is established by a different mechanism from other Drosophila epithelia, but more akin to that of vertebrate epithelia.

## Author summary

The *Drosophila* midgut is lined by a single-layered epithelium that acts as a barrier to the environment while allowing for nutrient uptake and related physiological processes. To fulfil these roles, midgut epithelial cells are highly polarised, with a pronounced asymmetric distribution of cellular components. Previous work in *Drosophila* revealed a conserved set of factors governing cell polarity, and it is thought that this network of proteins underlies all examples of polarity in *Drosophila* (and other organisms). Here, we demonstrate that the epithelial cells in the *Drosophila* midgut are not polarised by these canonical polarity factors but instead rely on the integrin adhesion complex. Thus, there are two types of epithelia in *Drosophila* that polarise using fundamentally different mechanisms. This diversity may reflect a difference in developmental origin (endodermal versus ectodermal), a difference in junctional arrangement, or the direction in which the respective cells polarise. Since knock-outs of canonical polarity factors often have little or no effect on epithelial polarity in vertebrate model systems, this diversity of polarity mechanisms might be conserved in other organisms.

## Introduction

Most animal organs and tissues are composed of epithelial cells that adhere laterally to each other to form sheets that act as barriers between compartments. The formation of epithelial monolayers depends on the coordinated polarisation of each cell along its apical–basal axis, and this polarity underlies all aspects of epithelial biology [1,2]. For example, the function of epithelia as barriers to fluids and pathogens depends on the correct positioning of the occluding cell–cell junctions (septate junctions [SJs] in invertebrates and tight junctions in vertebrates), whereas the adhesion between cells depends on the lateral localisation of cadherin-dependent adherens junctions (AJs).

Much of our understanding of how epithelial cells polarise comes from studies of *Drosophila melanogaster* that have identified a conserved set of epithelial polarity factors that define different cortical domains along the apical–basal axis of the cell. The apical domain is specified by the transmembrane protein Crumbs, the adaptor protein Stardust, and the Par-6/atypical protein kinase C (aPKC) complex; the boundary between the apical and lateral domains is defined by Bazooka (Baz, Par-3 in other organisms), which positions the apical-most lateral junction; and the rest of the lateral domain is marked by Scribbled (Scrib), Discs large (Dlg), and Lethal (2) giant larvae (Lgl) [3]. Null mutations in any of these factors disrupt epithelial polarity in the primary epithelium that forms from the cellular blastoderm of the *Drosophila* embryo and gives rise to most of the structures of the larva and adult [4–11]. Similarly, loss of any of these genes disrupts the secondary epithelium formed by the follicle cells that surround the developing oocyte [12–14]. In each tissue, Baz seems to play a pivotal role in positioning the apical AJs and in localising the apical factors, which then exclude Baz from the apical domain [15–19]. The identity of the apical and lateral domains is then maintained by mutual antagonism between apical and lateral factors [20,21]. The requirement for some of these factors becomes less stringent in polarised epithelia as they mature. For example, Crumbs is particularly important in epithelial tissues that are remodelling their cell junctions as they undergo morphogenetic rearrangements, and Scrib, Dlg, and Lgl are not required to maintain polarity in mid-embryogenesis, as the Yurt group of lateral proteins takes over the antagonism of the apical factors, although Scrib and Dlg are still required for the formation of the SJs [20–24].

Epithelial cells are thought to have evolved at the origin of multicellularity, as cells first started to adhere to each other to form sheets, suggesting that apical–basal polarity is an ancient invention that is controlled by a conserved mechanism [25]. In support of this view, all of the canonical epithelial factors in *Drosophila* are conserved in vertebrates and localise to the equivalent cortical domains [26–33]. Knock-downs or knock-outs of some of these factors impair polarity in certain contexts, such as in *Xenopus* embryonic blastomeres and in some cultured cell lines [34–37]. In most cases, however, knock-outs of canonical polarity factors have little or no effect on polarity or cause unrelated phenotypes. For example, mice homozygous for a null mutation in *PAR-3* die in mid-gestation from a heart defect caused by a failure of the epicardial cell migration, but other embryonic epithelia appear to form normally [38]. Similarly, knock-out of *PAR-3* in mouse mammary stem cells disrupts the morphogenesis of the mammary gland but not the ability of the cells to polarise [39,40]. Finally, Scrib functions as a planar cell polarity gene in mice but has no obvious effect on apical–basal polarity [41]. Although this lack of polarity phenotypes in mammals could be a result of redundancy between paralogues, it raises the possibility that at least some vertebrate epithelia polarise by different mechanisms from the model *Drosophila* epithelia. In support of this view, many mammalian epithelia require integrin adhesion to the basement membrane to orient their polarity, whereas the well-characterised *Drosophila* epithelia do not [42–44]. Furthermore, vertebrate epithelial cells have an inverted arrangement of junctions compared to insect epithelia: the apical junction in vertebrates is the occluding, tight junction, which forms at the apical/lateral boundary above a lateral zonula adherens (ZA), whereas insect cells form lateral SJs, below an apical ZA [45–47].

**Fig 1:**
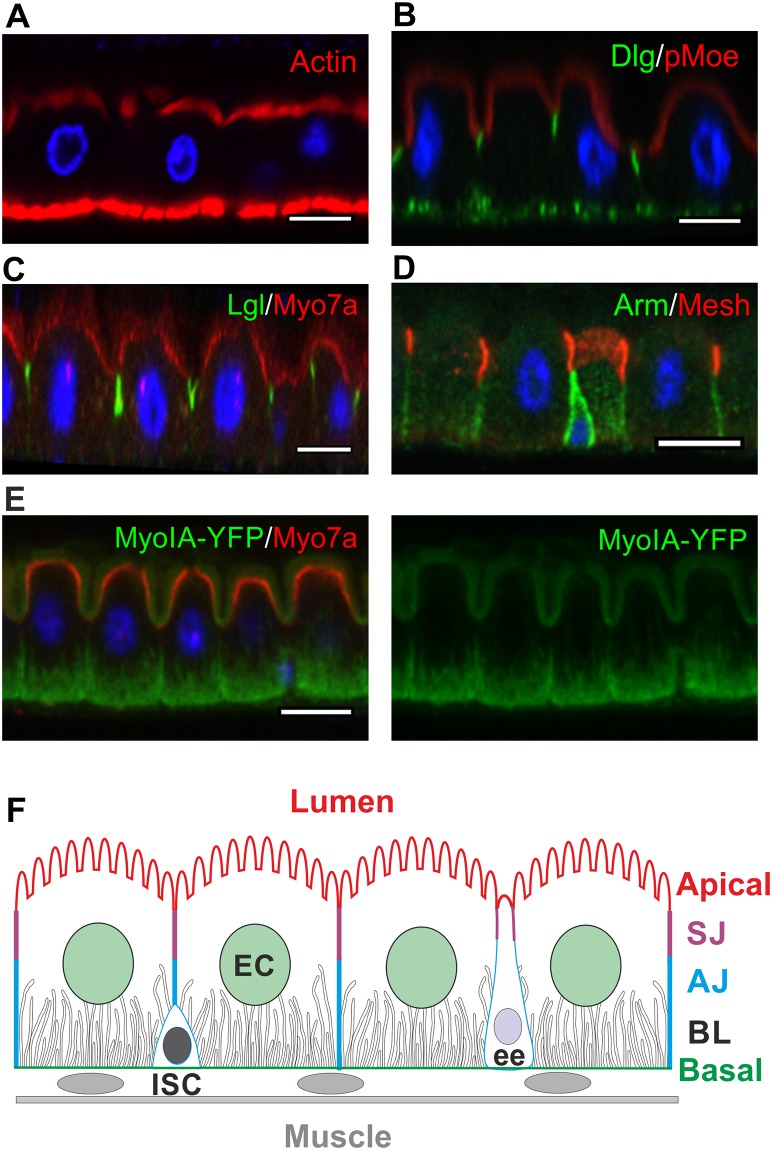
*Drosophila* midgut epithelial cells have a reversed arrangement of intercellular junctions compared to other *Drosophila* epithelia. All micrographs in this and succeeding figures show apical–basal confocal sections of the adult *Drosophila* midgut epithelium, with apical on top. (A–E) The adult *Drosophila* midgut epithelium stained for the following: (A) F-actin (phalloidin; red) and DNA (DAPI; blue), revealing the apical brush border. The strong basal signal corresponds to the visceral musculature. (B) pMoe (red), Dlg (green), and DNA (DAPI; blue). (C) Lgl (green), Myo7a (red), and DNA (DAPI, blue). The signal detected by the anti-Myo7a antibody in EC nuclei is unspecific, as it is also present in *ck*^13^ (Myo7a) mutant clones (S1G Fig). (D) The AJ component Armadillo (green) and the SJ component Mesh (red), revealing the organization of intercellular junctions in the midgut epithelium. (E) Myo31DF/MyoIA (green) and Myo7a (red). Myo31DF/MyoIA is mainly localised to the apical brush border and the BL, whereas Myo7a is localised to the apical domain, slightly below the brush border. (F) Diagram of the midgut epithelium, which is mainly composed of ECs with a lower frequency of ee cells, both of which turn over in the adult and are replaced by the progeny of basal ISCs. The diagram shows the localisation of the SJs above the AJs and the BL. Scale bars, 10 μm. AJ, adherens junction; Arm, Armadillo; BL, basal labyrinth; Dlg, Discs large; EC, enterocyte; ee, enteroendocrine cell; ISC, intestinal stem cell; Lgl, Lethal (2) giant larvae; MyoIA, Myosin IA; Myo7a, Myosin 7a; Myo31DF, Myosin 31DF; pMoe, phospho-Moesin; SJ, septate junction; YFP, yellow fluorescent protein.

The possibility that the polarisation of some epithelia is independent of the canonical polarity system prompted us to ask if all *Drosophila* epithelia polarise in the same way. We therefore examined polarity in the *Drosophila* adult midgut epithelium, which is mainly absorptive rather than secretory and is endodermal in origin, unlike the well-characterised epithelia, which are secretory and arise from the ectoderm or mesoderm [48]. The adult midgut is a homeostatic tissue in which basal intestinal stem cells (ISCs in Fig 1F) divide to produce new cells that integrate into the epithelium to replace dying enterocytes (ECs), which are shed into the gut lumen. This has the advantage that one can generate homozygous mutant stem cell clones in heterozygotes at the late pupal or adult stage to produce clonal patches of mutant ECs in the adult midgut without disrupting the development of the tissue. Our results reveal that the polarisation of the midgut epithelium does not require any of the canonical polarity factors and has several features in common with vertebrate epithelia, making it a useful model for investigating alternative polarity pathways.

## Results

The midgut is a typical epithelium with an apical brush border marked by F-actin (Fig 1A) and phospho-Moesin (pMoe) (Fig 1B) as well as Myosin IA (Fig 1E) and an apical domain marked by Myosin 7a(Fig 1C). However, our analysis led us to rediscover an interesting property of this epithelium: the smooth SJs, which form the occluding barrier to paracellular diffusion, form at the apical side of the lateral domain, above AJs, which are diffusely distributed over the lateral domain [49–52] (Fig 1D and 1F). This is the opposite way around compared to other *Drosophila* epithelia, in which the AJs condense to form a ZA around the apical margin of the cell, with the SJs, if present, positioned more basally in the lateral membrane. The organisation of junctions in the midgut therefore resembles the junctional arrangement in mammals, in which the occluding tight junctions form above the AJ.

In secretory epithelia, the Crumbs/Stardust complex defines the apical and marginal region and anchors the Par-6/aPKC complex in this domain [5,9,53]. Apical Crb and aPKC then exclude Baz/Par-3 to define the apical/lateral boundary by positioning the apical AJs [17–19]. This raises the question of whether these factors mark the same positions or the same structures when the junctions are reversed in the midgut.

**Fig 2:**
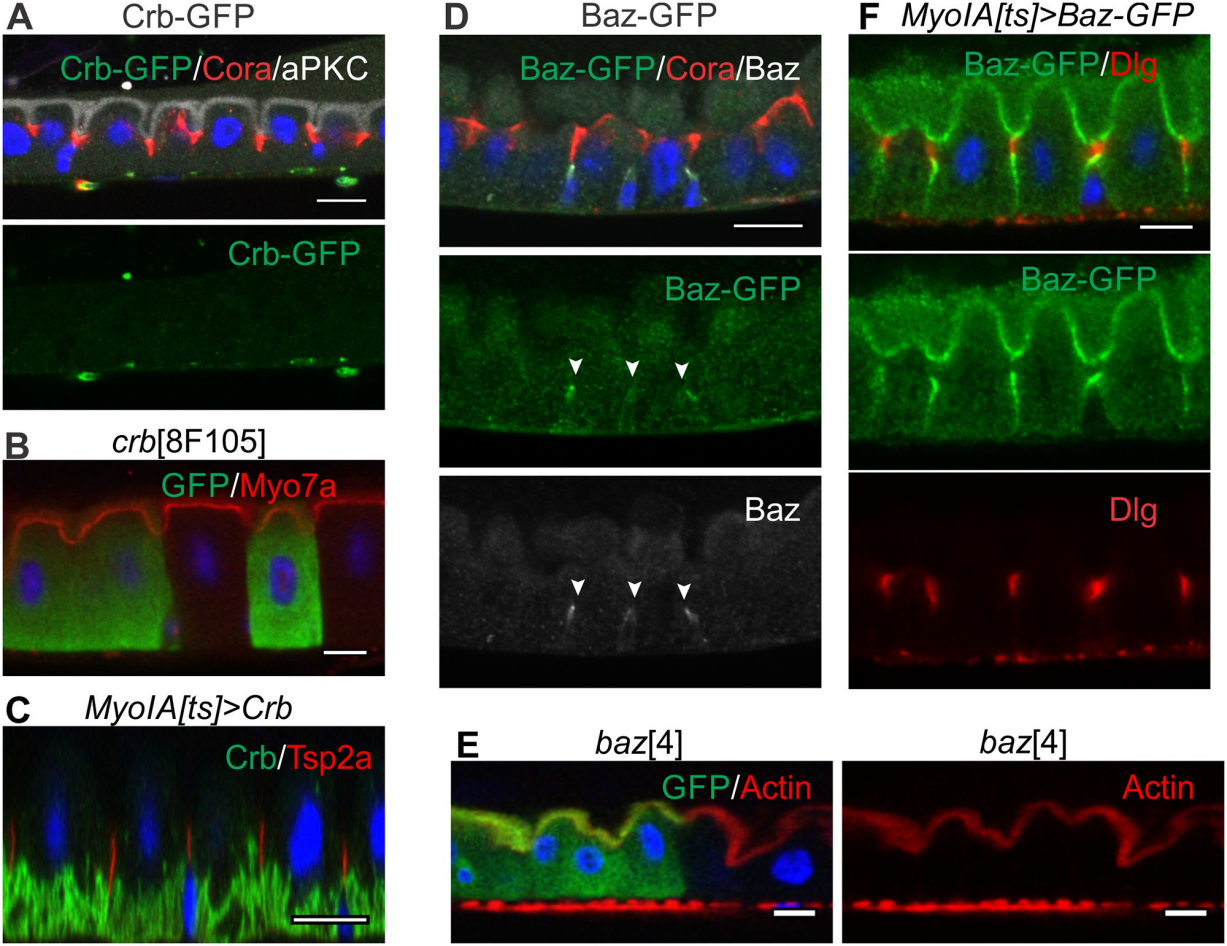
Crb and Baz are neither detectably expressed nor required for apical–basal polarity in adult midgut ECs. (A) Adult midgut epithelium from a female carrying a GFP protein trap insertion in the endogenous Crb locus stained with anti-GFP (green). Crb-GFP is not detectable in the adult midgut epithelium. Cora (red) and aPKC (white) are also shown. (B) ECs homozygous for *crb*^8F105^ (marked by GFP, green). The apical domain forms normally as revealed by the apical localization of Myo7a (red). (C) Conditional mis-expression of UAS-Crb in adult midgut ECs using MyoIA-GAL4; tubP-GAL80^ts^. Crb (green) localises to the BL but does not perturb SJ formation as revealed by Tsp2a (red). (D) Adult midgut epithelium expressing Baz-GFP under endogenous control (protein trap line), stained with anti-GFP (green) and anti-Baz (white). Baz/Baz-GFP is not detectable in ECs but is expressed in ISCs, in which it localises apically (white arrowhead). Cora (red) labels the SJ. (E) ECs homozygous mutant for *baz*^4^ (marked by GFP, green). The apical brush border forms normally as revealed by staining for F-actin with phalloidin (red). (F) Ectopic expression of UAS-Baz-GFP in adult midgut ECs using MyoIA-GAL4; tubP-GAL80^ts^ has no effect on the formation of the apical SJ marked by Dlg (red). Baz-GFP localises to the apical domain and AJs (green). Scale bars, 10 μm. AJ, adherens junction; aPKC, atypical protein kinase C; Baz, Bazooka; BL, basal labyrinth; Cora, Coracle; Crb, Crumbs; Dlg, Discs large; EC, enterocyte; GFP, green fluorescent protein; MyoIA, Myosin IA; Myo7a, Myosin 7a; SJ, septate junction; Tsp2a, Tetraspanin 2a; tubP, tubulin promoter; UAS, Upstream Activation Sequence.

Crumbs is not detectable in the adult midgut epithelium, as has previously been observed in the embryo [4] (Fig 2A). We used the mosaic analysis with a repressible cell marker (MARCM) technique [54] to generate positively marked clones in the adult midgut epithelium for null mutations in *crumbs* or *stardust* (*crb*^8F–105^, *crb*^11A22^; *sdt*^k85^). The adults were then dissected 8 to 10 days after clone induction to allow the stem cells, which divide about once a day, to go through multiple divisions because this rules out any perdurance of wild-type proteins expressed before the clones were induced. However, clones of *crb* and *sdt* null mutations give no obvious phenotypes (Fig 2B, S1A, S1D and S1E Fig). Overexpression of Crumbs expands the apical domain in other *Drosophila* epithelia [55]. By contrast, ectopic expression of Crumbs in the adult midgut epithelium does not affect EC polarity and the Crumbs protein does not localise apically, concentrating instead in the basal labyrinth (BL), an extensive set of tubular membrane invaginations from the EC basal surface (Fig 2C). Baz/Par-3 is also not detectable in ECs, although it is expressed in the ISCs, in which it localises apically as previously reported [56] (Fig 2D). MARCM clones homozygous for *baz* null alleles (*baz*^815–8^; *baz*^4^) occur at a similar frequency to wild-type clones and contain a normal number of cells, arguing against a role for Baz in asymmetric stem cell division, consistent with the view that these divisions are random and largely symmetric [57]. More importantly, *baz* null mutant ECs integrate into the epithelium and develop normal apical–basal polarity, in contrast to other epithelia in *Drosophila* (Fig 2E, S1B, S1C and S1F Fig). Baz-green fluorescent protein (GFP) localises apically and to the AJs when ectopically expressed in the midgut epithelium, consistent with binding to the Par-6/aPKC complex and to E-cadherin complexes (Fig 2F). However, ectopic Baz expression has no effect on the polarity of the ECs or on the formation of an apical SJ.

**Fig 3:**
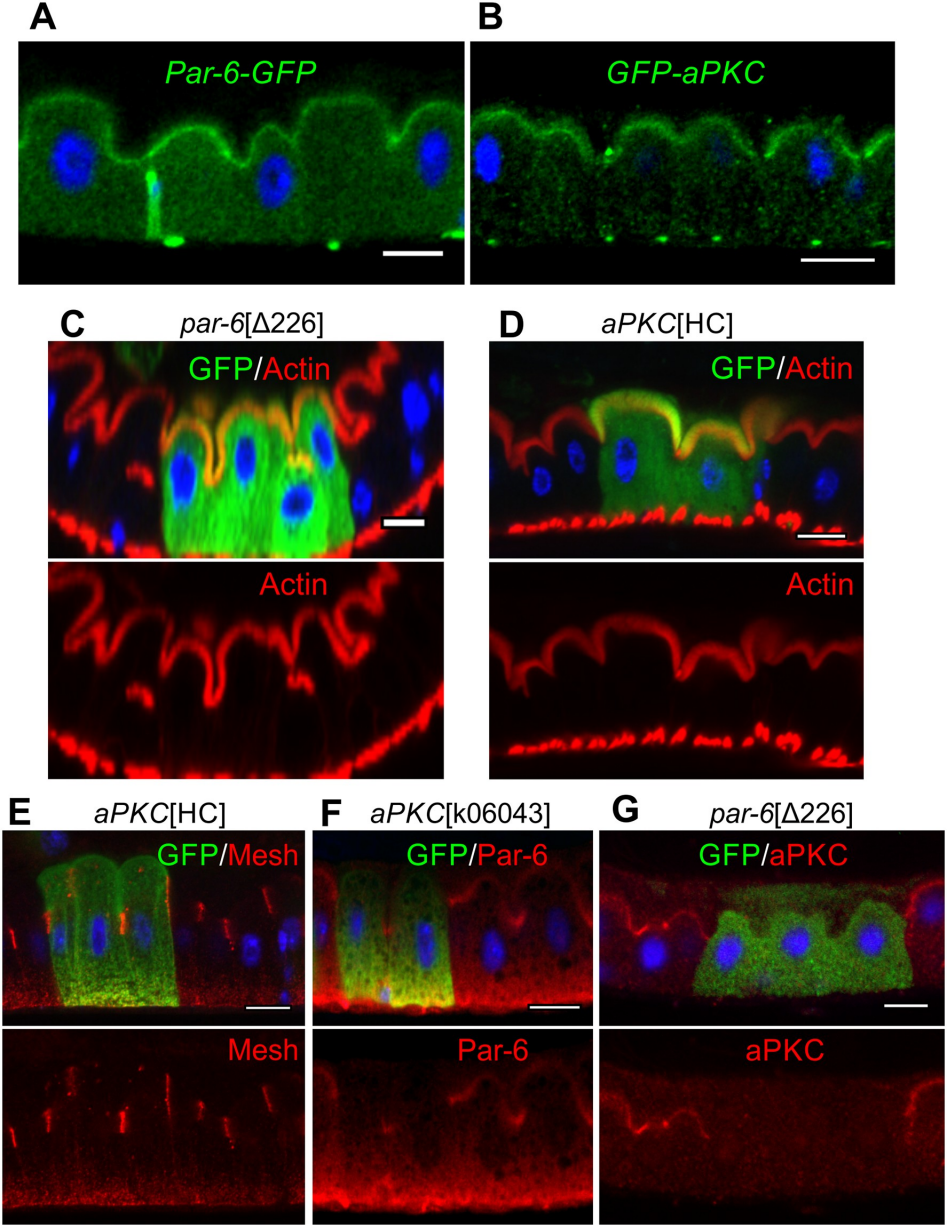
aPKC and Par-6 are not required for EC polarity. Subcellular localisation of endogenously tagged Par-6–GFP (A) and GFP-aPKC (B), as revealed by anti-GFP staining (green). MARCM clones (marked by GFP, green) homozygous mutant for *par-6*^Δ226^ (C) and *aPKC*^HC^ (D, E) show normal apical actin brush borders (F-actin in red) and SJ localisation (E; Mesh, red). The apical localisation of Par-6 (red) is lost in *aPKC*^K06043^ MARCM clones (marked by GFP, green) (F) as is the apical localisation of aPKC (red) in *par6*^Δ226^ MARCM clones (marked by GFP, green) (G). Scale bars, 10 μm. aPKC, atypical protein kinase C; EC, enterocyte; GFP, green fluorescent protein; MARCM, mosaic analysis with a repressible cell marker; SJ, septate junction.

Both aPKC and Par-6 are expressed in the midgut and localise apically, as they do in all other epithelia (Fig 3A and 3B). In most polarised cells, the apical localisation of the Par-6/aPKC complex depends on Baz/Par-3, and in epithelia, this also requires Crumbs and Stardust [2]. Consistent with our observation that these proteins are absent from ECs, neither Baz nor Crb are required for the localisation of Par-6, indicating that the latter must be targeted apically by a distinct mechanism (S1A and S1B Fig). Surprisingly, the apical domain forms normally in *par-6*^Δ226^ and *aPKC*^k06043^ mutant clones, and the morphology of the cells is unaffected (Fig 3C, S1H and S1J Fig). Although *aPKC*^k06043^ is considered a null allele, the corresponding P element insertion does not disrupt the shorter isoforms of aPKC. Thus, it is conceivable that some aPKC activity remains in *aPKC*^k06043^ homozygous mutant cells. We therefore used CRISPR to generate a complete null, *aPKC*^HC^, a frameshift mutation resulting in a premature stop codon and a truncation of aPKC at amino acid 409, which is located in the middle of the kinase domain (S1L Fig). Homozygous *aPKC*^HC^ clones also show no phenotype, forming normal actin-rich brush borders and SJs, confirming that aPKC is dispensable for EC polarity (Fig 3D and 3E). Nevertheless, Par-6 is lost from the apical domain in *aPKC*^k06043^ clones and aPKC is not apical in *par-6*^Δ226^ clones, showing that their localisations are interdependent (Fig 3F and 3G). Thus, the Par-6/aPKC complex still marks the apical domain of the midgut epithelium but is not required for its formation or maintenance.

**Fig 4:**
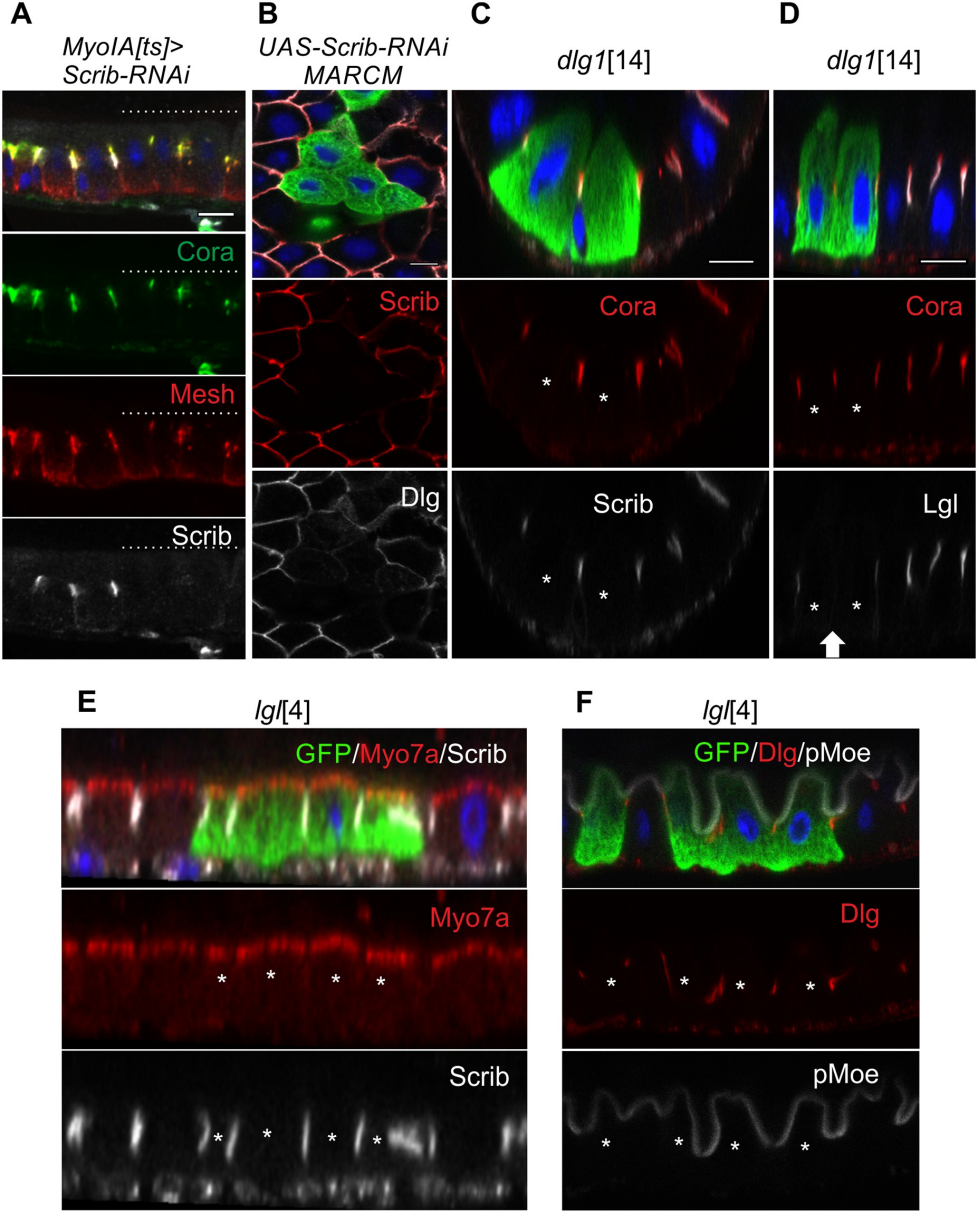
Scrib, Dlg, and Lgl localise to SJs but are not required for SJ formation or apical–basal polarity. (A) Mosaic knock-down of Scrib by RNAi in adult ECs. The SJ markers Cora (green) and Mesh (red) localise normally in cells depleted of Scrib (white). The ECs lacking Scrib are indicated by dashed lines above. (B) Dlg (red) does not localise to the SJs in Scrib–RNAi MARCM clones (marked by GFP, green). (C) Cora (red) and Scrib (white) localise to the SJs in *dlg1*^14^ MARCM clones (marked by GFP, green). (D) Lgl does not localise to the SJ between *dlg1*^14^ mutant cells (marked by GFP, green). The white arrow indicates the SJ between the *dlg1*^14^ mutant cells. (E) Myo7a (red) and Scrib (white) localise normally to the apical cortex and SJs respectively in *lgl*^4^ MARCM clones (marked by GFP, green). (F) Dlg (red) and pMoe (white) localise normally in *lgl*^4^ mutant cells (marked by GFP, green). White asterisks mark the mutant cells. Scale bars, 10 μm. Dlg, Discs large; EC, enterocyte; GFP, green fluorescent protein; Lgl, Lethal (2) giant larvae; MARCM, mosaic analysis with a repressible cell marker; Myo7a, Myosin 7a; pMoe, phospho-Moesin; RNAi, RNA intereference; Scrib, Scribbled; SJ, septate junction; UAS, Upstream Activation Sequence.

**Fig 5:**
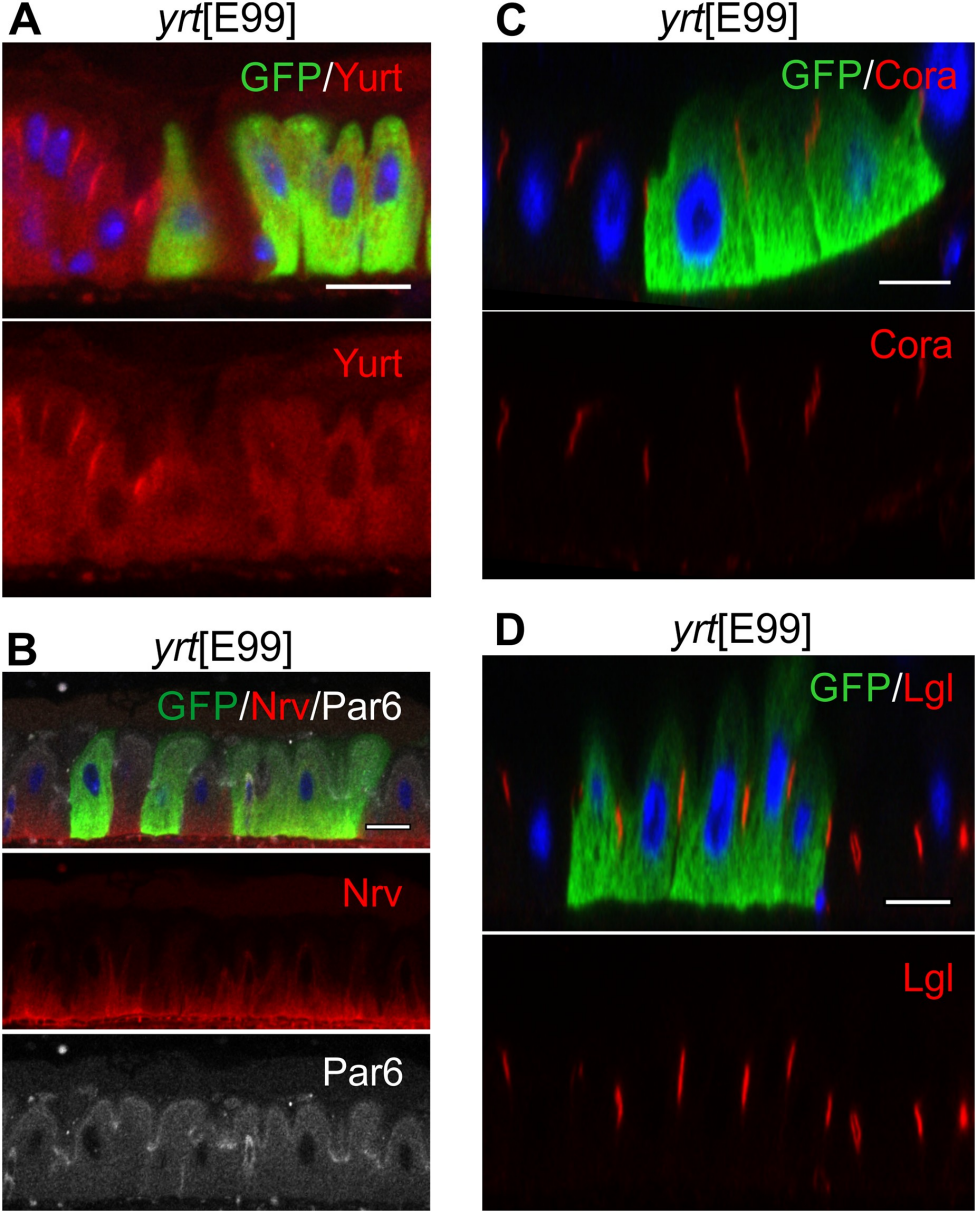
Yurt is not required for EC polarisation. (A) Yurt (red) localises to SJs and is not detectable in ECs mutant for the loss-of-function allele *yrt*[E99] (marked by GFP, green). (B) In *yurt* mutant clones (marked by GFP, green), Par-6 (white) still localises to apical domain and Na^+^ K^+^ ATPase (stained with the pan-Na–pump β-subunit (Nrv) [62] antibody Nrv5F7 (red)) still localises to BL. (C) and (D) Yurt is not required for SJ formation, as Cora (C) (red) and Lgl (D) (red) still localise to SJ in *yurt* mutant clones (marked by GFP, green). Scale bars, 10 μm. BL, basal labyrinth; EC, enterocyte; GFP, green fluorescent protein; Lgl, Lethal (2) giant larvae; Nrv, Nervana; SJ, septate junction.

The lateral polarity factors Scrib, Dlg, and Lgl are all expressed in the midgut and colocalise with each other to the SJs, marked by the conserved SJ component, Coracle [58] (Fig 1B and 1C, S2A Fig). Since the SJs form at the apical side of the lateral membrane in the midgut, in the position occupied by the AJs in other *Drosophila* epithelia, these proteins mark a conserved structure rather than a conserved position. The lateral epithelial polarity factors are required for the formation of pleated SJs in the embryo [20,21,59]. However, the smooth SJs form normally in *scrib*, *dlg*, and *lgl* mutant clones or when these factors are depleted by RNA interference (RNAi) (Fig 4A and 4C, S2B Fig). The apical domain is also unaffected in *scrib*, *dlg*, and *lgl* mutant or knock-down cells, in contrast to other epithelia in which apical factors are mislocalised to the basolateral domain (Fig 4E and 4F, S2C and S2D Fig). In stage 13 embryos, the Yurt complex excludes apical factors from the lateral membrane in place of Scrib, Dlg, and Lgl [23,24]. We therefore also examined the role of Yurt in the midgut. Yurt localises to the SJs, as it does in the embryo, but *yurt* mutant clones still polarise normally and form SJs that recruit Lgl (Fig 5). Finally, we examined Par-1, which localises laterally in other epithelia in which it plays a role in localising AJs through phosphorylation of Baz and in organising the microtubule cytoskeleton [60,61]. Par-1 is expressed in the ISCs, in which it localises laterally but is not detectable in the ECs. Consistent with its lack of expression, *par-1*^9^ mutant ECs show normal apical–basal polarity (S3A and S3B Fig). Thus, all the canonical epithelial polarity factors are dispensable for the polarisation of the midgut epithelium, even though Par-6, aPKC, Scrib, Dlg, Lgl, and Yurt are expressed and localise to equivalent positions to secretory epithelia.

The relationships between the lateral factors has been difficult to assess in other epithelia because mutants in *scrib*, *dlg*, and *lgl* give rise to round, unpolarised cells without an identifiable lateral domain [11]. We took advantage of the normal EC polarisation in these mutants to investigate the interdependence of their recruitment to the SJs. Neither Dlg nor Lgl are recruited to SJs in cells depleted of Scrib by RNAi (Fig 4B and S2D Fig). Scrib localises normally in *dlg* mutant clones, whereas both Scrib and Dlg localise normally to the SJs in *lgl* mutant clones (Fig 4C–4F). Thus, there is a simple hierarchical relationship between these factors in the midgut epithelium, in which Scrib is required to recruit Dlg, which is needed for Lgl localisation.

**Fig 6:**
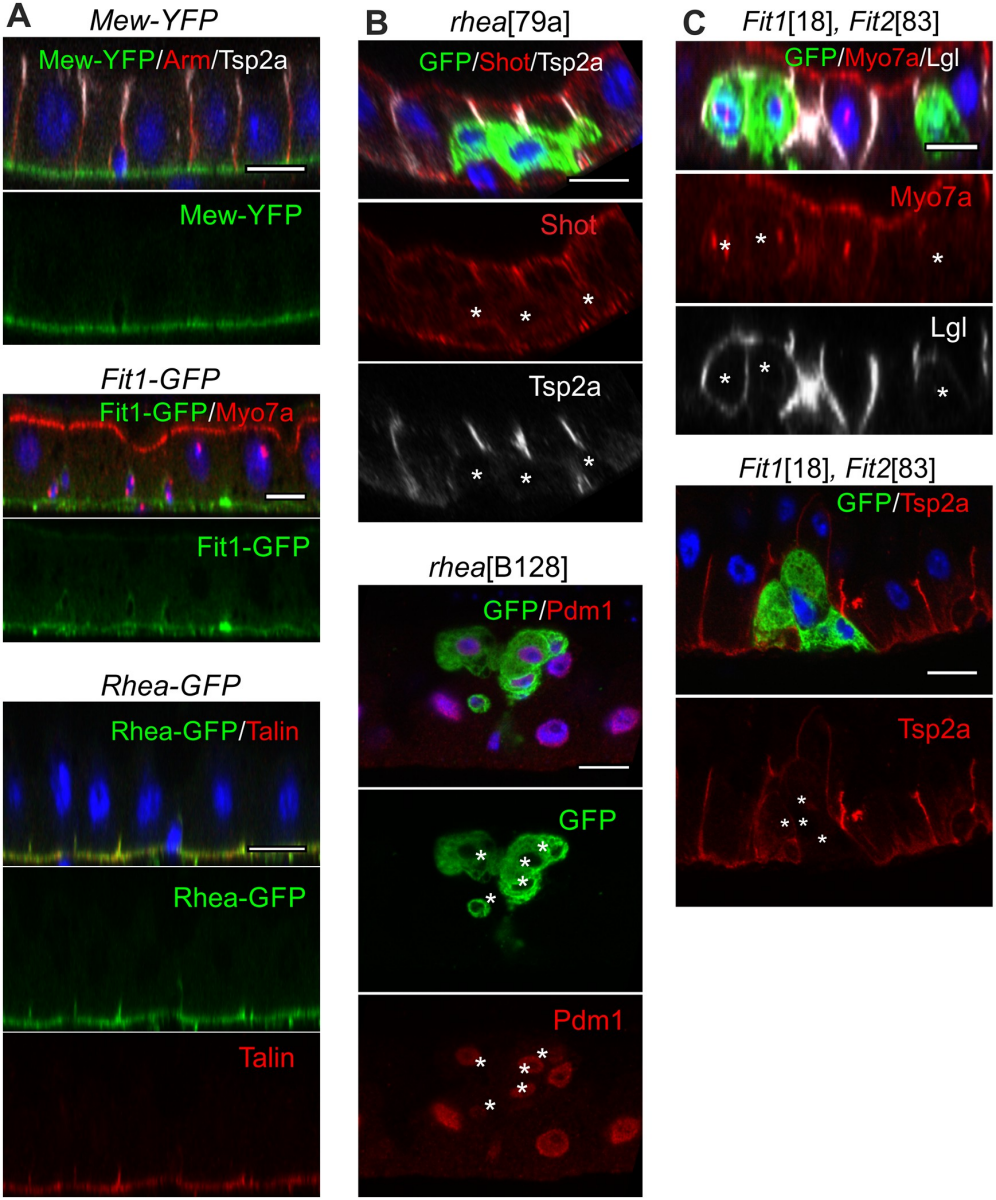
The integrin adhesion complex is required for EC polarisation and integration. (A) The α-integrin Mew and the cytoplasmic adaptor proteins of the integrin adhesion complex, Talin (Rhea) and Fermitin (Fit, also known as Kindlin), localise to the basal surface of the midgut epithelium. Mew-YFP, Rhea-GFP (protein trap insertions), and Fit1-GFP (a genomic fosmid construct) [66] were detected with an anti-GFP antibody (green), whereas the subcellular localization of Talin was also revealed with an anti-Talin antibody (red). (B) *rhea*^79a^ mutant cells (marked by GFP, green) detach from the basement membrane and fail to polarise. Shot (red) is apically enriched in neighbouring wild-type ECs but is not localised in *rhea* mutant cells, which fail to form SJs marked by Tsp2a (white). Most *rhea*^B128^ mutant cells (marked by GFP, green) express Pdm1 (red), a marker for differentiating ECs. (C) *Fit1*^18^ *Fit2*^83^ double mutant clones show a similar phenotype: Myo7a is not enriched apically (red), Lgl (white) spreads around the whole plasma membrane, and SJs fail to form as shown by the loss of Tsp2a localization (red). White asterisks * mark the mutant clones. Scale bars, 10 μm. EC, enterocyte; GFP, green fluorescent protein; Lgl, Lethal (2) giant larvae; Myo7a, Myosin 7a; SJ, septate junction; Tsp2a, Tetraspanin 2a; YFP, yellow fluorescent protein.

The surprising observation that none of the classical epithelial polarity factors are required for the apical–basal polarisation of the midgut epithelium raises the question of how polarity is generated and maintained. Given the similar junctional arrangement to mammalian epithelia, we addressed whether polarity in midgut ECs depends on integrin-dependent adhesion to the extracellular matrix, as it does in several mammalian epithelia [42–44]. Components of the integrin adhesion complex, such as the α-integrin Mew and the essential cytoplasmic adaptor proteins Talin (*Drosophila* Rhea) [63] and Kindlin (*Drosophila* Fit 1 [64]; Fit 2 is not detectable expressed in the midgut) are highly localised to the basal side of the midgut epithelium (Fig 6A). The expression of two α-integrins and two β-integrins in the midgut complicates the genetic analysis of their function, so we focused on the cytoplasmic components of the integrin adhesion complex. Clones of cells homozygous for null alleles of *rhea* (*rhea*^79a^ and *rhea*^B128^) detach from the basement membrane and fail to polarise, forming irregularly shaped cells that do not form SJs or an apical domain (Fig 6B). Most *rhea* mutant cells remain within the epithelial layer, below the SJs of the wild-type cells, probably because they do not form SJs themselves (Fig 6B and S4A Fig). Despite their inability to polarise and integrate into the epithelium, the mutant cells still appear to differentiate: they become polyploid, the size of their nuclei is not significantly different from that of wild-type cells (*rhea* nuclear long axis: 6.0 μm ± 1.0 μm versus heterozygous cells: 6.9 μm ± 0.9 μm), and they express the marker for differentiating ECs, Pdm1 [65] (Fig 6B). Cells mutant for both *Fit1* and *Fit2* show a similar phenotype to Talin mutants. Mutant cells have normal nuclear dimensions (*Fit1 Fit2* nuclear long axis: 6.6 μm ± 1.2 μm versus heterozygous cells: 6.9 μm ± 1.0 μm) and express Pdm1 but fail to localise apical markers or form SJs (Fig 6C and S4B Fig).

**Fig 7:**
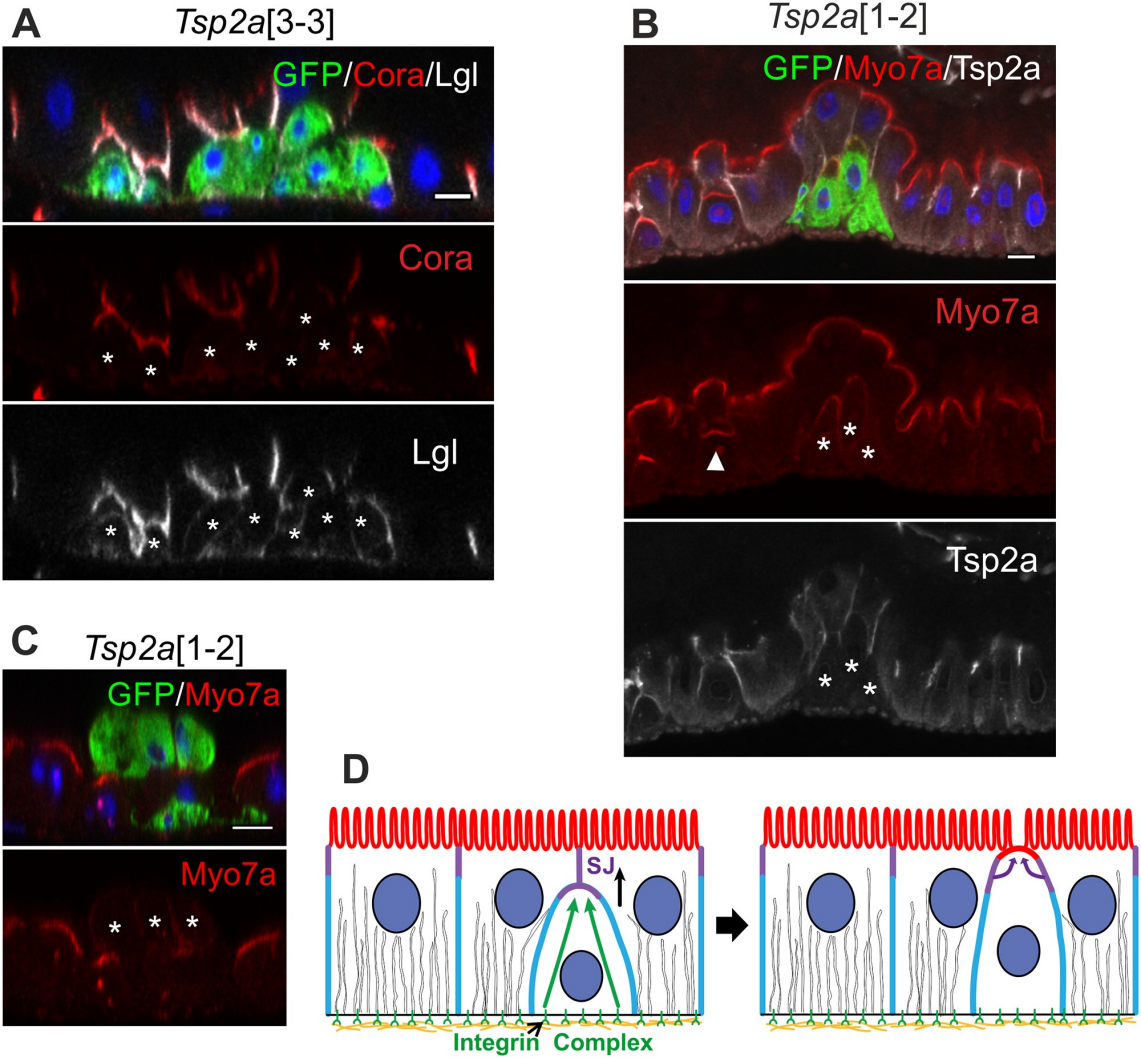
SJ proteins are required for EC polarisation and integration. (A) *Tsp2a* mutant clones (marked by GFP, green) fail to integrate into the epithelium, forming basal clusters that lack SJs, as indicated by the loss of Cora (red) localization and diffuse Lgl staining (white). (B) and (C) Neither *Tsp2a* mutant cells (marked by GFP, green) that cluster on the basal side (B) or that face the lumen of the gut (C) form an apical domain, as shown by the lack of apical Myo7a (red) enrichment. (D) Model of the steps in EC polarisation: adhesion to the basement membrane is required for SJ formation, which in turn is necessary for the formation of an apical domain. White asterisks * mark the mutant clones. White arrowhead in (B) marks the integrating EC. Scale bars, 10 μm. Cora, Coracle; EC, enterocyte; GFP, green fluorescent protein; Lgl, Lethal (2) giant larvae; Myo7a; Myosin 7a; SJ, septate junction; Tsp2a, Tetraspanin 2a.

The ISCs lie beneath the epithelium and differentiating ECs must therefore integrate into the epithelium from the basal side, inserting between the SJs of the flanking ECs while maintaining an intact barrier. We therefore examined the effects of mutations in the core SJ components Tsp2a and Mesh [51,52]. More than 90% of mutant cells fail to integrate through the SJs of the neighbouring wild-type cells, and the clones form clusters on the basal side of the epithelium (Fig 7A, S3A and S4C Figs). Nevertheless, the mutant cells still appear to differentiate normally, as shown by their nuclear size (Tsp2a nuclear long axis: 6.0 μm ± 0.9 μm versus heterozygous cells: 6.6 μm ± 0.8 μm) and Pdm1 expression (S4D Fig). Wild-type cells start to form an apical domain as they integrate, before they have a free apical surface, as shown by the enrichment of apical components such as Myo7a (arrowhead in Fig 7B). By contrast, apical markers only weakly localise in cells mutant for SJ components and never form a clear apical domain, even if the cells are extruded from the apical side of the epithelium (Fig 7B and 7C). Thus, the midgut epithelium appears to polarise in a basal to apical manner, in which adhesion to the ECM is required for the formation of the SJs and the SJs are needed for the formation of the apical domain (Fig 7D).

## Discussion

Our results reveal that the intestinal epithelium polarises by a fundamentally different mechanism from other *Drosophila* epithelia, as none of the classical epithelial polarity genes are required for its apical–basal polarisation. This cannot be attributed to redundancy between paralogues, as might be the case in vertebrates, because all of the polarity factors are single-copy genes in *Drosophila*. Thus, our observations provide strong evidence against the idea that there is a universal system for polarising epithelial cells. Nevertheless, a core set of the polarity factors (Par-6, aPKC, Scrib, Dlg, and Lgl) is expressed in the midgut epithelium, and these proteins localise to the equivalent positions to other *Drosophila* epithelia. Thus, they may still serve important functions in the midgut epithelium that are not essential for the overall apical–basal polarisation of the cells.

Given that all other epithelia in *Drosophila* use the canonical polarity pathway, our observations raise the question of why the midgut epithelium is different. This is unlikely to reflect the fact that the midgut is absorptive rather than secretory, as secretory cells in the midgut, such as the enteroendocrine (ee) cells and the acid-secreting copper cells, polarise in the same way as the ECs (S1J and S1K Fig). One key difference between the midgut epithelium and other epithelia is that it is the only epithelial tissue of endodermal origin in the fly, whereas all other epithelia are ectodermal or mesodermal. Thus, it is possible that endodermal epithelia are intrinsically different in the way that they polarise. In support of this view, it has recently been found that PAR-3, PAR-6, and aPKC are degraded in the invaginating endomesoderm of the Cnidarian *Nematostella vectensis* and are not required for this tissue to form an epithelium [67]. Thus, the difference between endodermal and ectodermal polarity systems may have evolved before the origin of Bilateria. The *Drosophila* midgut arises from the cellular blastoderm of the embryo and initially polarises in the same way as other embryonic epithelia before it undergoes an epithelial-to-mesenchymal transition (EMT) under the control of the endodermal GATA family transcription factor, Serpent [68]. Serpent drives EMT at least in part by inhibiting the transcription of *crumbs* and *stardust* and might therefore contribute to the switch in polarity mechanisms. However, Serpent is turned off as the midgut primordium migrates and is not expressed when the cells reform an epithelium to generate the midgut tube. Indeed, continued expression of Serpent blocks the cells from re-epithelising after migration. Thus, a pulse of Serpent expression may trigger the switch in polarity mechanisms, perhaps through downstream transcription factors, but Serpent itself prevents epithelial polarisation.

A second important difference between the adult midgut epithelium and other epithelia in *Drosophila* is its reversed arrangement of occluding junctions and AJs, with apical SJs forming above lateral AJs. In other *Drosophila* epithelia, Baz plays a key role in concentrating the AJs at the apical margin of the lateral membrane to form the ZA [69]. Thus, the absence of Baz in midgut ECs may contribute to the absence of an apical ZA. This cannot be the only factor making the midgut different, however, as ectopic expression of Baz in midgut ECs does not alter the position of the SJs. One reason why the SJs may have evolved to form above the AJs in the midgut is that this places the barrier to paracellular diffusion apically, thereby preventing the contents of the gut lumen from accessing the lateral sides of the cell, which is presumably important because the gut is full of digestive enzymes and potential pathogens. Other *Drosophila* epithelia that face the external environment—such as the epidermis, trachea, foregut, and hindgut—secrete an impermeable cuticle, which provides a protective covering to prevent pathogens from accessing the cell surface [70]. The development of the typical arthropod cuticular exoskeleton may therefore have freed these epithelia from the need to place their occluding junctions apically, allowing the apical positioning of the ZA.

The third important difference between the midgut epithelium and other epithelia is that the ECs polarise in a basal-to-apical direction as they integrate into the epithelium, whereas all other *Drosophila* epithelia polarise in an apical-to-basal direction. For example, the primary embryonic epithelium forms during cellularisation, as the furrow canals grow in from the apical surface of the embryo between the nuclei, while the follicle cells polarise in response to an apical cue from the germ line [14,71]. Thus, it is possible that the mechanism by which cells polarise depends on the order in which they generate the basal, lateral, and apical domains. Since the midgut is the only endodermal epithelium in *Drosophila*, the only epithelium with apical SJs, and the only epithelium that polarises from basal to apical, it is not possible to determine which of these characteristics underlies its alternative mechanism of cell polarisation, and this will require analysis in other organisms with a greater diversity of epithelial cell types.

Whatever the reason for the alternative polarity mechanism in the *Drosophila* midgut epithelium, its polarity is much more similar to that of well-characterised vertebrate epithelia than other *Drosophila* epithelia. Firstly, the midgut and vertebrate epithelia have apical occluding junctions above lateral AJs, whereas other *Drosophila* epithelia have a reversed arrangement of junctions. Secondly, the midgut epithelium does not require the canonical epithelial polarity factors that are essential in other *Drosophila* epithelia, and this also seems to be the case in some vertebrate epithelia, although this may be due to redundancy between paralogues. Thirdly, the midgut epithelium and a number of vertebrate epithelia depend on signals from the integrin adhesion complex to polarise correctly [42–44]. These similarities suggest that the *Drosophila* midgut epithelium may prove a better in vivo model for at least some types of vertebrate epithelia. It will therefore be important to determine whether vertebrates also contain distinct types of epithelia whose polarity is controlled by different factors.

## Materials and methods

### *D*. *melanogaster* genetics

*w*^1118^ or *y*^2^ or *OreR* flies were used as wild type unless otherwise specified. Other stocks used in this study were as follows:

Fluorescently tagged protein lines: EGFP-aPKC (this paper), Par6-EGFP [72], Mew-YFP [73] (Kyoto DGRC #115524), Baz-EGFP [74] (Bloomington #51572), Myo31DF-YFP [73] (Kyoto DGRC #115611), Crb-EGFP [75] (gift from Y. Hong, University of Pittsburgh, United States of America), Fit1-EGFP [66] (gift from B. Klapholz and N. Brown, Department of Physiology, Development and Neuroscience, University of Cambridge, United Kingdom), Rhea-GFP (Bloomington #39650), Par-1-GFP [61,76].
Mutant stocks: *aPKC*^k06043^ [6], *aPKC*^HC^ (this paper), *par6*^Δ226^ [77], *dlg1*^A^ (Bloomington #57086), *dlg1*^14^ [59], *lgl*^4^ [78], *baz*^815-8^ [79], *baz*^4^ [8], *crb*^8F105^ [4], *crb*^11A22^ [4], *yurt*^E99^ [80], *ck*^13^ (Bloomington #5259), *Tsp2A*^1-2^, *Tsp2A*^3-3^, *Tsp2A*^2-9^ [52] (gift from M. Furuse, Kobe University, Japan), *mesh*^f04955^ [51] (Kyoto DGRC #114660), *Fit1*^18^, *Fit2*^83^, *Fit1*^18^*Fit2*^83^ [64], *rhea*^79a^ [63], *rhea*^B28^, *rhea*^B128^ [64], *ilk*^54^ [81] (gifts from B. Klapholz and N. Brown, Department of Physiology, Development and Neuroscience, University of Cambridge, UK), *par-1*^9^ (a missense mutation that changes Valine 279 to Aspartate adjacent to the ATP binding pocket of the Par-1 kinase domain. This allele was characterised by Teresa Niccoli and behaves like a null mutation genetically).
UAS responder lines: UAS-Scrib–RNAi (Bloomington #35748), UAS-Crb (Bloomington #5544), UAS-Baz-GFP [17].

The following stocks were used to generate (positively labelled) MARCM [54] clones:

MARCM FRTG13: y w, UAS-mCD8::GFP, Act5C-GAL4, hsFLP[1]; FRTG13 tubP-GAL80.
MARCM FRT82B: y w, UAS-mCD8::GFP, Act5C-GAL4, hsFLP[1];; FRT82B tubP-GAL80.
MARCM FRT19A: w, hsFLP, tubP-GAL80, FRT19A;; tubP-GAL4, UAS-mCD8::GFP/TM3, Sb.
MARCM FRT2A: hsFLP[1]; tubP-GAL4, UAS-mCD8::GFP/CyO, GFP; FRT2A tubP-GAL80 (gift from B. Klapholz and N. Brown).
MARCM FRT40A: y w, UAS-mCD8::GFP, Act5C-GAL4, hsFLP[1]; FRT40A tubP-GAL80.

Negatively marked clones on the X chromosome were generated using the following stock:

y w His2Av::GFP hsFLP[12] FRT19A/FM7a (Bloomington #32045).
Clones mutant for *mesh* were generated using the following stock: esg-GAL4, UAS-FLP, tubP-GAL80[ts]/CyO; FRT82B nlsGFP (referred to as esg > FLP[ts] in S4C Fig; gift from G. Kolahgar, Department of Physiology, Development and Neuroscience, University of Cambridge, UK).
UAS-Crb and Baz-GFP were expressed in the adult midgut epithelium using the following driver line: y w; MyoIA-GAL4, tubP-GAL80[ts] (referred to as MyoIA[ts] in Figs 2C, 2F and 4A and S2D Fig; gift from G. Kolahgar).

### Stock maintenance

Standard procedures were used for *Drosophila* husbandry and experiments. Flies were reared on standard fly food supplemented with live yeast at 25 °C. For the conditional expression of UAS responder constructs (e.g., RNAi) in adult flies, parental flies were crossed at 18 °C and the resulting offspring reared at the same temperature until eclosion. Adult offspring were collected for 3 days and then transferred to 29 °C to inactivate the temperature-sensitive GAL80 protein. To generate MARCM or GFP-negative clones, flies were crossed at 25 °C and the resulting offspring subjected to heat shocks either as larvae (from L2 until eclosion) or as adults (5–9 days after eclosion). Heat shocks were performed at 37 °C for 1 h (twice daily). Flies were transferred to fresh food vials every 2–3 days and kept at 25 °C for at least 9 days after the last heat shock to ensure that all wild-type gene products from the heterozygous progenitor cells had turned over. For this study, all (midgut) samples were obtained from adult female flies.

### Formaldehyde fixation

Samples were dissected in PBS and fixed with 8% formaldehyde (in PBS containing 0.1% Triton X-100) for 10 min at room temperature. Following several washes with PBS supplemented with 0.1% Triton X-100 (washing buffer), samples were incubated in PBS containing 3% normal goat serum (NGS, Stratech Scientific Ltd, Cat. #005-000-121; concentration of stock solution: 10 mg/ml) and 0.1% Triton X-100 (blocking buffer) for 30 min. This fixation method was only used for samples in which F-actin was stained with fluorescently labelled phalloidin, as phalloidin staining is incompatible with heat fixation.

### Heat fixation

The heat fixation protocol is based on a heat–methanol fixation method used for *Drosophila* embryos [82]. Samples were dissected in PBS, transferred to a wire mesh basket, and fixed in hot 1X TSS buffer (0.03% Triton X-100, 4 g/L NaCl; 95 °C) for 3 s before being transferred to ice-cold 1X TSS buffer and chilled for at least 1 min. Subsequently, samples were transferred to washing buffer and processed for immunofluorescence stainings.

### Immunofluorescence staining

After blocking, samples were incubated with the appropriate primary antibody/antibodies diluted in blocking buffer at 4 °C overnight. Following several washes, samples were incubated with the appropriate secondary antibody/antibodies either at room temperature for 2 h or at 4 °C overnight. Samples were then washed several times in washing buffer and mounted in Vectashield containing DAPI (Vector Laboratories) on borosilicate glass slides (No. 1.5, VWR International). All antibodies used in this study were tested for specificity using clonal analysis (MARCM) or RNAi.

Primary antibodies:

Mouse monoclonal antibodies: anti-Dlg (4F3), anti-Cora (c615.16), anti-αSpec (3A9), anti-Arm (N2 7A1), anti-Talin (A22A, E16B), anti-Pros (MR1A), anti-Crb (Cq4), anti-Nrv (Nrv5F7), anti-Mys (CF.6G11). All monoclonal antibodies were obtained from the Developmental Studies Hybridoma Bank and used at 1:100 dilution.

Rabbit polyclonal antibodies: anti-pEzrin (NEB Cat. #3726S, 1:200 dilution); anti-Lgl (Santa Cruz Biotechnoloy Inc., d-300, Cat. #SC98260, 1:200 dilution); anti-aPKC (Santa Cruz Biotechnoloy Inc., Cat #SC216, 1:100 dilution); anti-β_H_Spec [83] (gift from C. Thomas, The Pennsylvania State University, USA, 1:1,000 dilution); anti-Baz [6] (gift from A. Wodarz, University of Cologne, Germany, 1:200 dilution); anti-Par6 [84] (gift from D. J. Montell, UCSB, USA, 1:500 dilution); anti-Mesh [51] and anti-Tsp2A [52] (gift from M. Furuse, 1:1,000 dilution); anti-Scrib [85] (gift from C. Q. Doe, University of Oregon, USA, 1:1,000 dilution); anti-Pdm1 [86] (gift from F. J. Diaz-Benjumea, Centre for Molecular Biology "Severo Ochoa" (CBMSO), Spain, 1:1,000 dilution); anti-Cno [87] (gift from M. Peifer, UNC, USA, 1:1,000 dilution).

Other antibodies used: chicken anti-GFP (Abcam, Cat. #ab13970, 1:1,000 dilution); guinea pig anti-Yurt [88] (gift from U. Tepass, University of Toronto, Canada, 1:1,000 dilution); guinea pig anti-Myo7a [89] (gift from D. Godt, University of Toronto, Canada, 1:1,000 dilution); guinea pig anti-Shot [90] (1:1,000 dilution); rat anti-Mesh [51] (gift from M. Furuse, 1:1,000 dilution).

Secondary antibodies:

Alexa Fluor secondary antibodies (Invitrogen) were used at a dilution of 1:1,000.

Alexa Fluor 488 goat anti-mouse (#A11029), Alexa Fluor 488 goat anti-rabbit (#A11034), Alexa Fluor 488 goat anti-guinea pig (#A11073), Alexa Fluor 488 goat anti-chicken IgY (#A11039), Alexa Fluor 555 goat anti-rat (#A21434), Alexa Fluor 555 goat anti-mouse (#A21422), Alexa Fluor 555 goat anti-rabbit (#A21428), Alexa Fluor 568 goat anti-guinea pig (#A11075), Alexa Fluor 647 goat anti-mouse (#A21236), Alexa Fluor 647 goat anti-rabbit (#A21245), Alexa Fluor 647 goat anti-rat (#A21247). Only cross-adsorbed secondary antibodies were used in this study to eliminate the risk of cross-reactivity.

F-Actin was stained with phalloidin conjugated to Rhodamine (Invitrogen, Cat. #R415, 1:500 dilution).

### Imaging

Images were collected on an Olympus IX81 (40× 1.35 NA Oil UPlanSApo, 60× 1.35 NA Oil UPlanSApo) using the Olympus FluoView software Version 3.1 and processed with Fiji (ImageJ).

### Generation of endogenous EGFP-aPKC

Endogenously tagged aPKC with EGFP fused to the N-terminus was generated by CRISPR-mediated homologous recombination. In vitro synthesised gRNA [91] to a CRISPR target approximately 60 nucleotides downstream from the *aPKC* start codon (target sequence GAATAGCGCCAGTATGAACATGG) and a plasmid donor containing the ORF of EGFP as well as appropriate homology arms (1.5 kb upstream and downstream) were coinjected into nos-Cas9–expressing embryos (Bloomington #54591; also known as CFD2) [92]. Single F0 flies were mated to *y w* flies and allowed to produce larvae before the parent was retrieved for PCR analysis. Progeny from F0 flies in which a recombination event occurred (as indicated by PCR) were further crossed and analysed to confirm integration. Several independent EGFP-aPKC lines were isolated. Recombinants carry the EGFP coding sequence inserted immediately downstream of the endogenous start codon and a short linker (amino acid sequence: Gly-Ser-Gly-Ser) between the coding sequence for EGFP and the coding sequence for aPKC. Homozygous flies are viable and healthy.

### Generation of *aPKC[HC]*

We used the CRISPR/Cas9 method [91] to generate a null allele of *aPKC*. sgRNA was in vitro transcribed from a DNA template created by PCR from two partially complementary primers: forward primer: 5′-GAAATTAATACGACTCACTATAggattacggcatgtgtaaggGTTTTAGAGCTAGAAATAGC-3′; reverse primer: 5′- AAAAGCACCGACTCGGTGCCACTTTTTCAAGTTGATAACGGACTAGCCTTATTTTAACTTGCTATTTCTAGCTCTAAAAC-3′. The sgRNA was injected into Act5c-Cas9 embryos [92]. Putative *aPKC* mutants in the progeny of the injected embryos were recovered, balanced, and sequenced. The *aPKC*^HC^ allele contains a small deletion around the CRISPR site, resulting in one missense mutation and a frameshift that leads to stop codon at amino acid 409 in the middle of the kinase domain, which is shared by all isoforms (S1L Fig). The *aPKC*^HC^ allele was subsequently recombined onto FRTG13 to generate MARCM clones. No aPKC protein was detectable by antibody staining in both midgut and follicle cell clones, and follicle cells homozygous for *aPKC*^HC^ display a phenotype that is indistinguishable from that observed in follicle cells homozygous for the *aPKC*^K06403^ allele.

### Quantification and statistical analysis

The proportions of *rhea*, *Fit*, and *Tsp2a* mutant cells inside the epithelial layer were calculated as follows: images were taken of different regions of several midguts containing MARCM clones stained with an apical marker. Cells that were above the neighbouring cells or had a clear apical domain were counted as ‘cells NOT inside the layer’, whereas cells without a detectable free apical surface were counted as ‘cells inside the layer’. Data were analysed with Graphpad Prism software. The graph in S4A Fig shows the average percent of cells inside the layer ± SD%.

The nuclear long axis of *rhea*, *Fit*, and *Tsp2a* mutant clones was manually measured in 20 random mutant cells (stained with DAPI, excluding obvious ISCs) and 20 surrounding heterozygous cells using Fiji.

### Reproducibility of experiments

All experiments were repeated multiple times with independent crosses: Baz-EGFP (4 independent experiments), EGFP-aPKC (9 independent experiments), Mew-YFP (5 independent experiments), Par6-EGFP (4 independent experiments), Crb-EGFP (5 independent experiments), Fit1-EGFP (4 independent experiments), Myo31DF-YFP (4 independent experiments), MyoIA[ts] > UAS-Scrib-RNAi (11 independent experiments), MyoIA[ts] > UAS-Crb (6 independent experiments), and MyoIA[ts] > UAS-Baz-GFP (3 independent experiments).

The phenotypes of homozygous mutant clones were analysed in multiple guts from independent experiments as follows: *baz*^4^ (13 independent experiments, 3,078 mutant cells analysed); *baz*^815-8^ (4 independent experiments, 734 cells analysed); *aPKC*^k06043^ (8 independent experiments, 3,681 mutant cells analysed); *aPKC*^HC^ (9 independent experiments, 15,984 mutant cells analysed); *par6*^Δ226^ (4 independent experiments, 2,558 mutant cells analysed); *crb*^11A22^ (4 independent experiments, 1,478 mutant cells analysed); *crb*^8F105^ (5 independent experiments, 3,288 mutant cells analysed); *lgl*^4^ (6 independent experiments, 6,790 mutant cells analysed); *dlg1*^14^ (5 independent experiments, 3,092 mutant cells analysed); *rhea*^79a^, rhea^B28^, *rhea*^B128^ (4 independent experiments for each genotype, 184 mutant cells analysed in total); *Fit1*^18^*Fit*2^83^ (7 independent experiments, 608 double mutant cells analysed); *Fit*1^18^ (4 independent experiments, 854 mutant cells analysed); *ilk*^54^ (5 independent experiments, 65 mutant cells analysed); *Tsp2a*^1-2^, *Tsp2a*^2-9^, and *Tsp2a*^3-3^ (7, 4, and 6 independent experiments, respectively; a total of 1,205 *Tsp2a* mutant cells were analysed); and *mesh*^f04955^ (5 independent experiments, 643 mutant cells analysed).

## Supporting information

S1 Fig Baz and Crb are not detectable in ECs and are not required for polarity. (A) and (B) Par-6 (red) localises normally to the apical surface of *crb*^8F105^ (A) and *baz*^4^ (B) mutant cells (marked by GFP, green). (C) *baz*^815–8^ mutant cells (marked by GFP, green) form a normal apical brush border as revealed by phalloidin staining of F-actin (red). (D) *crb*^11A22^ MARCM clones (marked by GFP, green) show normal Mesh (red) and Cno (white) localisation. (E) *sdt*^K85^ MARCM clones (marked by GFP, green) show normal Arm (red) and Lgl (white) localisation. (F) *baz*^4^ MARCM clones (marked by GFP, green) show normal Mesh (red) and Cno (white) localization. (G) Unspecific signals of Myo7a staining (red) in nuclei are present in *ck*^13^ MARCM clones (marked by GFP, green). (H) *aPKC*^k06043^ MARCM clones show normal Cora (red) at SJs and βHSpec (white) at the apical domain. (I) *aPKC*^k06043^ MARCM clones show normal αSpec (red) localization at the cortex. (J) The copper cell region has similar polarity to other regions of the midgut, with pMoe (red) at the apical domain, although folded inside, and Dlg (green) at apical SJs. (K) ee cells, which are labelled with Pros (green in nucleus), have Tsp2a (red) at the apical SJ and Arm (green) at the lateral AJ. (L) Schematic genomic map showing the *aPKC*^HC^ allele and corresponding protein sequence. Scale bars, 10 μm. AJ, adherens junction; aPKC, atypical protein kinase C; Arm, Armadillo; αSpec, α-Spectrin; Baz, Bazooka; βHSpec, βH-Spectrin; Cno, Canoe; Crb, Crumbs; Dlg, Discs large; EC, enterocyte; ee, enteroendocrine; GFP, green fluorescent protein; Lgl, Lethal (2) giant larvae; MARCM, mosaic analysis with a repressible cell marker; Myo7a, Myosin 7a; pMoe, phospho-Moesin; Pros, Prospero; SJ, septate junction.
(TIF)

S2 Fig Loss of Scrib or Dlg has no effect on apical domain formation in ECs. (A) Scrib (red) localises to the EC SJs, marked by Cora (green). (B) Mesh (red) localises normally to the SJs of *lgl*^4^ mutant cells (marked by GFP, green). (C) *dlg1*^14^ MARCM clones (marked by GFP, green) show normal apical localization of α-Spectrin (red) and β_H_-Spectrin (white). (D) RNAi knock-down of Scrib in adult midgut ECs has no effect on the subcellular localization of Myo7a (red) but disrupts Dlg (green) and Lgl (white) localisation to the SJs. Scale bar, 10 μm. Dlg, Discs large; EC, enterocyte; GFP, green fluorescent protein; Lgl, Lethal (2) giant larvae; MARCM, Mosaic analysis with a repressible cell marker; Myo7a, Myosin 7a; RNAi, RNA interference; Scrib, Scribbled; SJ, septate junction.
(TIF)

S3 Fig Par-1 is not required for EC polarity. (A) Par-1 is not detectable in ECs, although it is expressed in ISCs, as revealed by staining for GFP (green) in a Par-1 GFP protein trap line. Dlg is in red and aPKC in white. (B) A MARCM clone of *par-1*^9^ (marked by GFP, green). The mutant cells form normal SJs marked by Lgl (red). Scale bar, 10 μm. aPKC, atypical protein kinase C; Dlg, Discs large; EC, enterocyte; GFP, green fluorescent protein; ISC, intestinal stem cell; Lgl, Lethal (2) giant larvae; MARCM, mosaic analysis with a repressible cell marker; SJ, septate junction.
(TIF)

S4 Fig Talin, Kindlin, and SJ components are required for EC polarity. (A) Most *rhea*, *Fit1*, *Fit1 Fit2*, and *Tsp2a* mutant cells remain inside the epithelia layer. The graph is based on the analysis of 299 cells in wild-type MARCM clones (13 images), 1,205 *Tsp2a* mutant cells (25 images from *Tsp2a*^1–2^, *Tsp2a* ^3–3^, and *Tsp2a* ^2–9^ clones), 608 *Fit1*^18^ *Fit2*^83^ (*Fit*^D^) double mutant cells (23 images), 854 *Fit1*^18^ mutant cells (24 images), and 184 *rhea* mutant cells (18 images from *rhea*^B28^, *rhea*^79a^, and *rhea* ^B128^). The underlying data can be found in S1 Data. (B) *Fit1*^18^ *Fit2*^83^ double mutant cells (marked by GFP, green) differentiate as ECs, as revealed by the expression of Pdm1 (white). Talin is in red. (C) A *mesh*^f04955^ mutant clone (marked by the loss of GFP) stained for α–Spectrin (red) and Mesh (white). (D) *Tsp2a*^1–2^ mutant cells (marked by GFP, green) differentiate as ECs, as revealed by the expression of Pdm1 (red). White asterisks * and lines mark the mutant clones. Scale bars, 10 μm. EC, enterocyte; GFP, green fluorescent protein; MARCM, mosaic analysis with a repressible cell marker; SJ, septate junction.
(TIF)

S1 Data Excel spreadsheet containing, in separate sheets, the underlying numerical data of S4A Fig. Images were taken of different regions of several midguts containing MARCM clones stained with an apical marker. Cells that were above the neighbouring cells or had a clear apical domain were counted as ‘cells NOT inside the layer’, whereas cells without a detectable free apical surface were counted as ‘cells inside the layer’. For each image, the percentage of clones inside the layer among all clones was calculated. Data were analysed with Graphpad Prism software. The graph in S4A Fig shows the average percent of cells inside the layer ± SD%.
(XLSX)

## Abbreviations

AJ: adherens junction
aPKC: atypical protein kinase C
Arm: Armadillo
Baz: Bazooka
BL: basal labyrinth
Cno: Canoe
Cora: Coracle
Crb: Crumbs
CRISPR: Clustered Regularly Interspaced Short Palindromic Repeats
Dlg: Discs large
EC: enterocyte
ee: enteroendocrine
EMT: epithelial to mesenchymal transition
GFP: green fluorescent protein
ISC: intestinal stem cell
Lgl: Lethal (2) giant larvae
MARCM: Mosaic analysis with a repressible cell marker
Myo31DF: Myosin31DF
Myo7a: Myosin 7a
MyoIA: Myosin IA
NGS: normal goat serum
Nrv: Nervana
pMoe: phospho-Moesin
Pros: Prospero
RNAi: RNA inteference
Scrib: Scribbled
SJ: septate junction
Tsp2a: Tetraspanin 2A
tubP: tubulin promoter
UAS: Upstream Activation Sequence
YFP: yellow fluorescent protein
ZA: zonula adherens
αSpec: α-Spectrin
βHSpec: βH-Spectrin
