# Supplementary figures and images for: An alternative mode of epithelial polarity in the Drosophila midgut
Source: PLoS Biol. 2018 Oct 19;16(10):e3000041. doi: 10.1371/journal.pbio.3000041 (PMC6209374; doi:10.1371/journal.pbio.3000041)

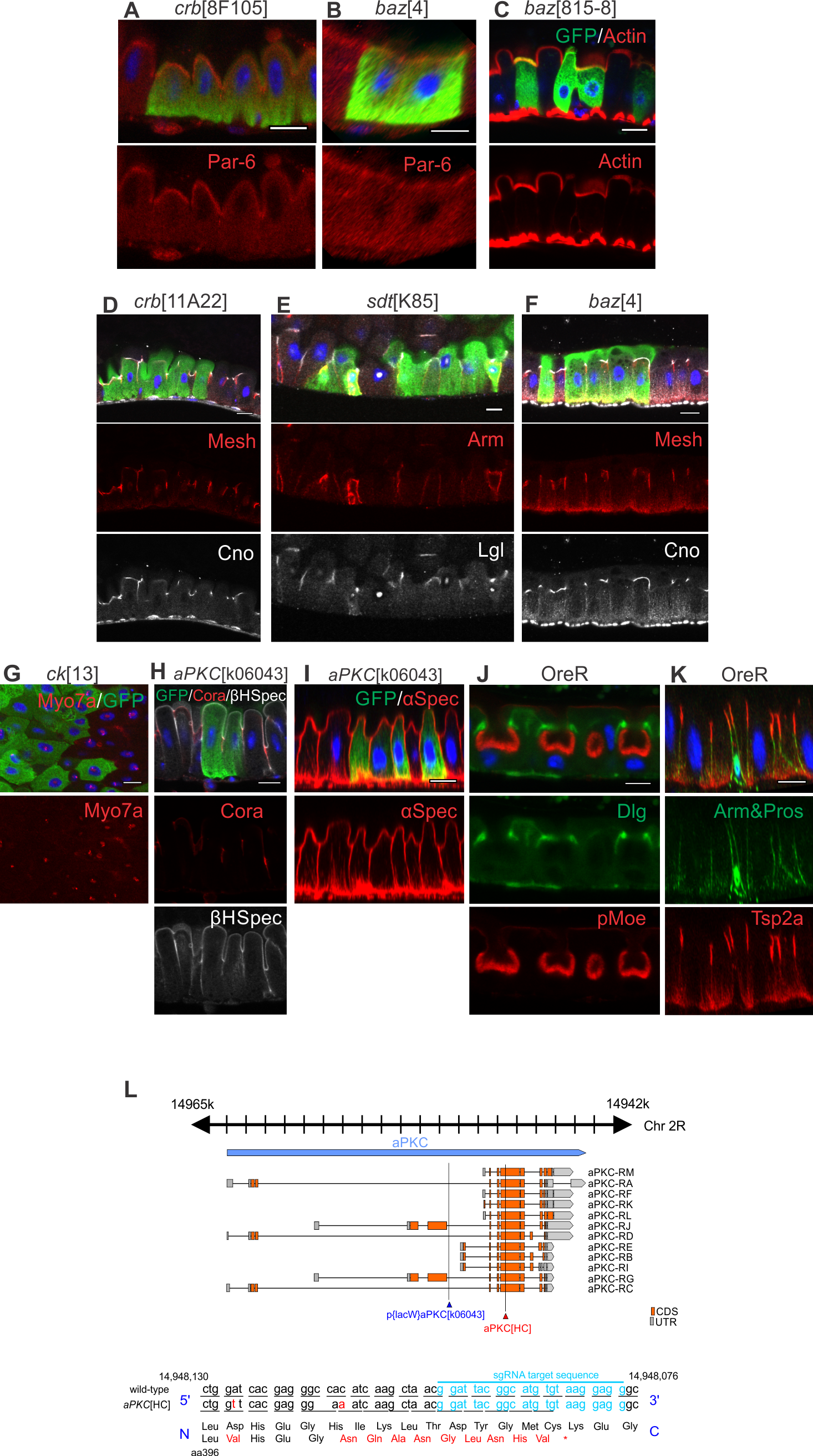

Supplement: S1 Fig — (A) and (B) Par-6 (red) localises normally to the apical surface of crb8F105 (A) and baz4 (B) mutant cells (marked by GFP, green). (C) baz815–8 mutant cells (marked by GFP, green) form a normal apical brush border as revealed by phalloidin staining of F-actin (red). (D) crb11A22 MARCM clones (marked by GFP, green) show normal Mesh (red) and Cno (white) localisation. (E) sdtK85 MARCM clones (marked by GFP, green) show normal Arm (red) and Lgl (white) localisation. (F) baz4 MARCM clones (marked by GFP, green) show normal Mesh (red) and Cno (white) localization. (G) Unspecific signals of Myo7a staining (red) in nuclei are present in ck13 MARCM clones (marked by GFP, green). (H) aPKCk06043 MARCM clones show normal Cora (red) at SJs and βHSpec (white) at the apical domain. (I) aPKCk06043 MARCM clones show normal αSpec (red) localization at the cortex. (J) The copper cell region has similar polarity to other regions of the midgut, with pMoe (red) at the apical domain, although folded inside, and Dlg (green) at apical SJs. (K) ee cells, which are labelled with Pros (green in nucleus), have Tsp2a (red) at the apical SJ and Arm (green) at the lateral AJ. (L) Schematic genomic map showing the aPKCHC allele and corresponding protein sequence. Scale bars, 10 μm. AJ, adherens junction; aPKC, atypical protein kinase C; Arm, Armadillo; αSpec, α-Spectrin; Baz, Bazooka; βHSpec, βH-Spectrin; Cno, Canoe; Crb, Crumbs; Dlg, Discs large; EC, enterocyte; ee, enteroendocrine; GFP, green fluorescent protein; Lgl, Lethal (2) giant larvae; MARCM, mosaic analysis with a repressible cell marker; Myo7a, Myosin 7a; pMoe, phospho-Moesin; Pros, Prospero; SJ, septate junction. (TIF) [file pbio.3000041.s001.tif]

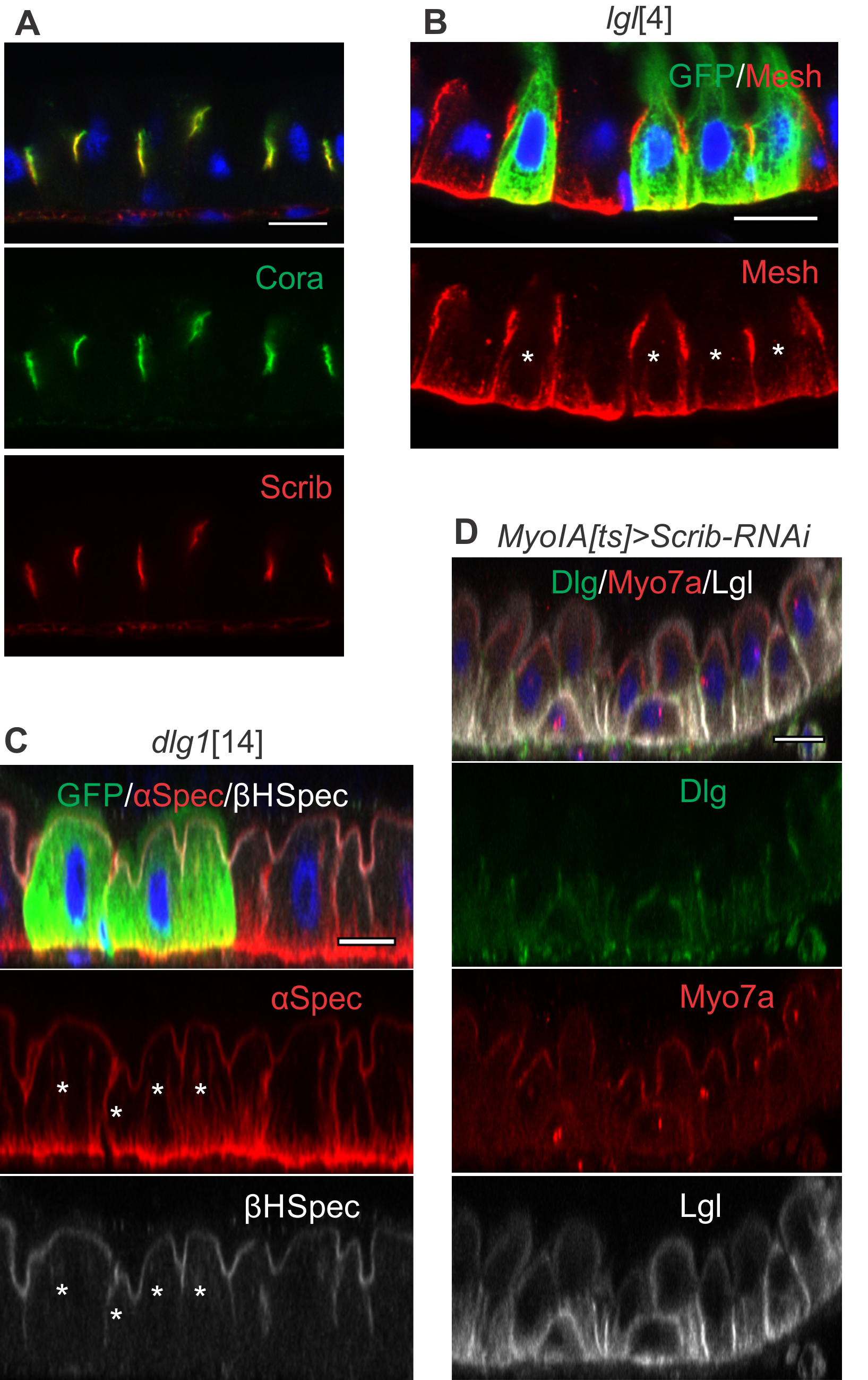

Supplement: S2 Fig — (A) Scrib (red) localises to the EC SJs, marked by Cora (green). (B) Mesh (red) localises normally to the SJs of lgl4 mutant cells (marked by GFP, green). (C) dlg114 MARCM clones (marked by GFP, green) show normal apical localization of α-Spectrin (red) and βH-Spectrin (white). (D) RNAi knock-down of Scrib in adult midgut ECs has no effect on the subcellular localization of Myo7a (red) but disrupts Dlg (green) and Lgl (white) localisation to the SJs. Scale bar, 10 μm. Dlg, Discs large; EC, enterocyte; GFP, green fluorescent protein; Lgl, Lethal (2) giant larvae; MARCM, Mosaic analysis with a repressible cell marker; Myo7a, Myosin 7a; RNAi, RNA interference; Scrib, Scribbled; SJ, septate junction. (TIF) [file pbio.3000041.s002.tif]

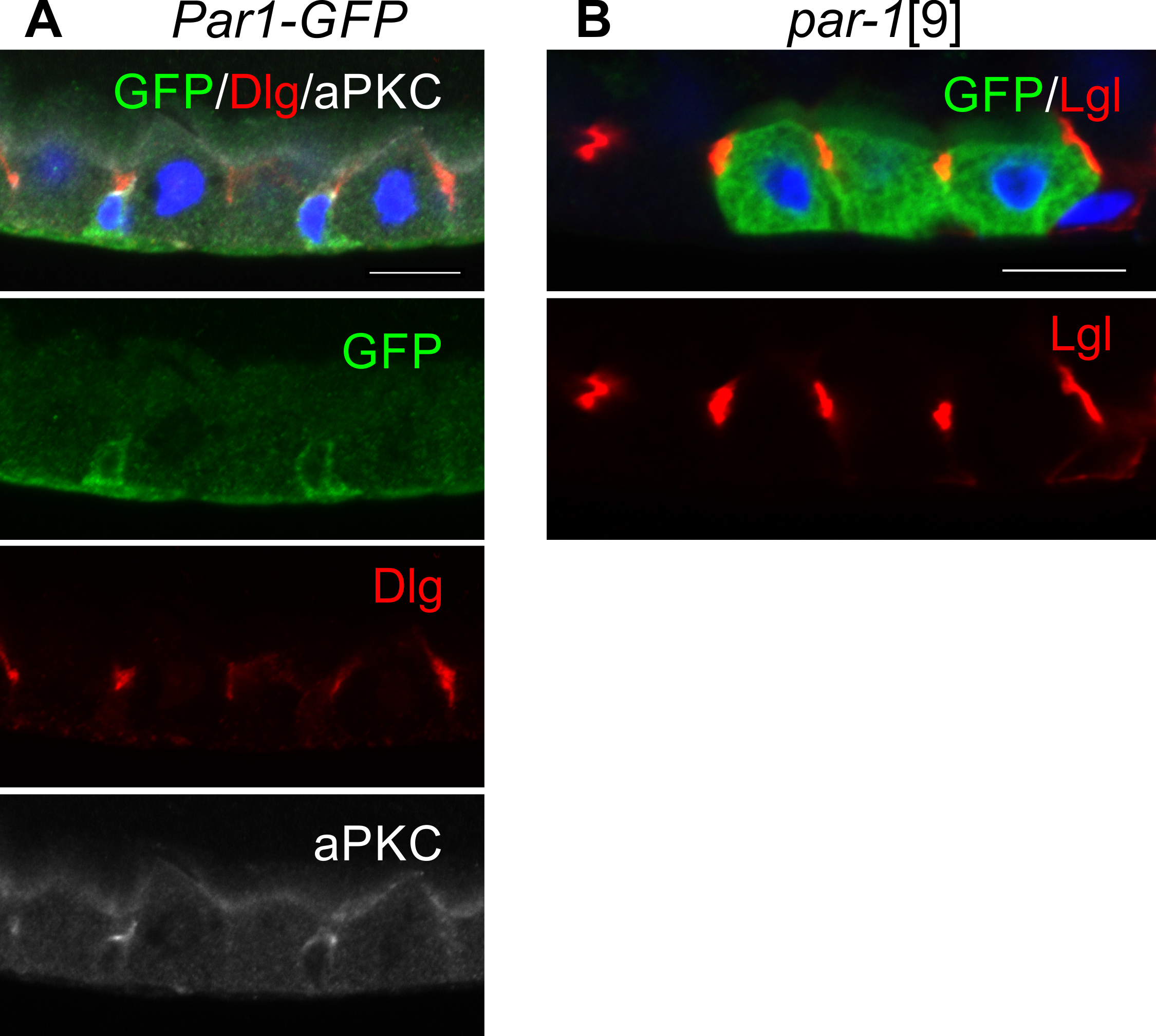

Supplement: S3 Fig — (A) Par-1 is not detectable in ECs, although it is expressed in ISCs, as revealed by staining for GFP (green) in a Par-1 GFP protein trap line. Dlg is in red and aPKC in white. (B) A MARCM clone of par-19 (marked by GFP, green). The mutant cells form normal SJs marked by Lgl (red). Scale bar, 10 μm. aPKC, atypical protein kinase C; Dlg, Discs large; EC, enterocyte; GFP, green fluorescent protein; ISC, intestinal stem cell; Lgl, Lethal (2) giant larvae; MARCM, mosaic analysis with a repressible cell marker; SJ, septate junction. (TIF) [file pbio.3000041.s003.tif]

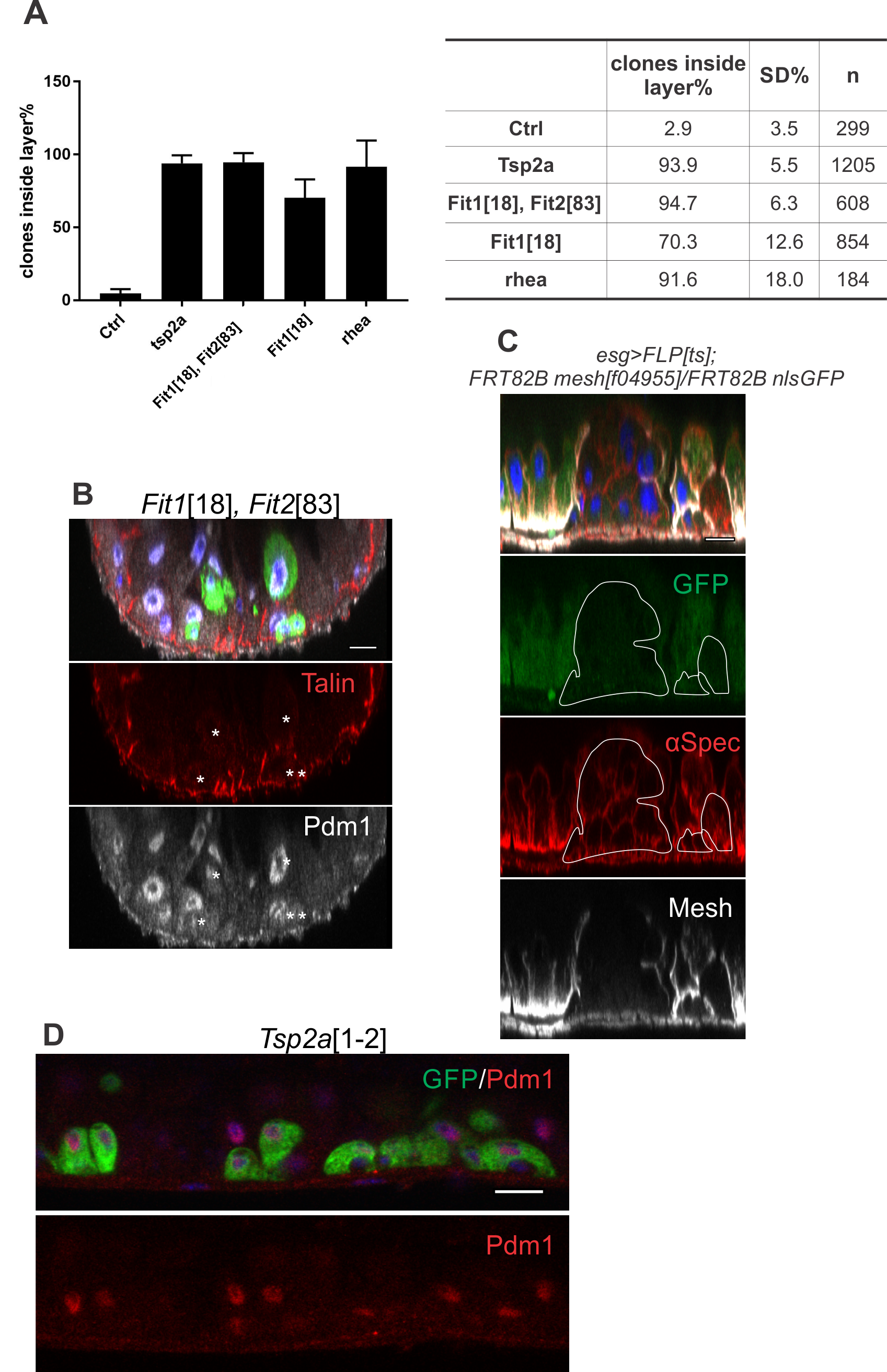

Supplement: S4 Fig — (A) Most rhea, Fit1, Fit1 Fit2, and Tsp2a mutant cells remain inside the epithelia layer. The graph is based on the analysis of 299 cells in wild-type MARCM clones (13 images), 1,205 Tsp2a mutant cells (25 images from Tsp2a1–2, Tsp2a 3–3, and Tsp2a 2–9 clones), 608 Fit118 Fit283 (FitD) double mutant cells (23 images), 854 Fit118 mutant cells (24 images), and 184 rhea mutant cells (18 images from rheaB28, rhea79a, and rhea B128). The underlying data can be found in S1 Data. (B) Fit118 Fit283 double mutant cells (marked by GFP, green) differentiate as ECs, as revealed by the expression of Pdm1 (white). Talin is in red. (C) A meshf04955 mutant clone (marked by the loss of GFP) stained for α–Spectrin (red) and Mesh (white). (D) Tsp2a1–2 mutant cells (marked by GFP, green) differentiate as ECs, as revealed by the expression of Pdm1 (red). White asterisks * and lines mark the mutant clones. Scale bars, 10 μm. EC, enterocyte; GFP, green fluorescent protein; MARCM, mosaic analysis with a repressible cell marker; SJ, septate junction. (TIF) [file pbio.3000041.s004.tif]
